# Moving Average Based Index for Judging the Peak of the COVID-19 Epidemic

**DOI:** 10.3390/ijerph17155288

**Published:** 2020-07-22

**Authors:** Yunting He, Xiaojin Wang, Hao He, Jing Zhai, Bingshun Wang

**Affiliations:** 1 School of Public Health, Shanghai Jiao Tong University School of Medicine, Shanghai 200025, China; heyunt1017@126.com (Y.H.); hezhihaomail@163.com (H.H.); zhaijianghe222@126.com (J.Z.); 2 Department of Biostatistics, Clinical Research Institute, Shanghai Jiao Tong University School of Medicine, Shanghai 200025, China; wangxiaojin106@sjtu.edu.cn

**Keywords:** novel coronavirus COVID-19, infectious diseases, moving average, epidemic peak, epidemic evaluation, epidemic

## Abstract

A pneumonia outbreak caused by a novel coronavirus (COVID-19) has spread around the world. A total of 2,314,621 laboratory-confirmed cases, including 157,847 deaths (6.8%) were reported globally by 20 April 2020. Common symptoms of COVID-19 pneumonia include fever, fatigue, and dry cough. Faced with such a sudden outbreak of emerging infectious disease, traditional models for predicting the peak of the epidemic often show inconsistent results. With the aim to timely judge the epidemic peak and provide support for decisions for resuming production and returning to normal life based on publicly reported data, we used a seven-day moving average of log-transformed daily new cases (LMA) to establish a new index named the “epidemic evaluation index” (EEI). We used SARS epidemic data from Hong Kong to verify the practicability of the new index, and then applied it to the COVID-19 epidemic analysis. The results showed that the epidemic peaked, respectively, on 9 February and 5 February 2020, in Hubei Province and other provinces in China. The proposed index can be applied for judging the epidemic peak. While the global COVID-19 epidemic reached its peak in the middle of April, the epidemic peaks in some countries have not yet appeared. Global and united efforts are still needed to eventually eliminate the epidemic.

## 1. Introduction

In late December 2019, an outbreak of an unexplained pneumonia occurred in Wuhan, Hubei Province, China, and subsequently evolved into a global pandemic. Presently, the pathogenic virus has been proven to be a novel coronavirus (COVID-19), which shares 79.5% sequence identity with SARS-CoV [1]. A total of 2,314,621 laboratory-confirmed cases, including 157,847 deaths (6.8%) have been reported globally by 20 April 2020 [2]. Common symptoms of COVID-19 infection include fever, fatigue, and dry cough. Some patients have experienced severe complications, including acute respiratory distress syndrome (ARDS), arrhythmia, and shock [3]. According to one study, the mean incubation period is three days [4]. Obviously, the COVID-19 epidemic can spread rapidly by human-to-human transmission, but the exact epidemiological characteristics and the specific modes of transmission remain partially unknown. The basic reproduction number (R0) was used to evaluate the transmission ability of COVID-19. Riou et al. estimated that the R0 of COVID-19 in January was about 2.2, suggesting the possibility for continuous human-to-human transmission [5]. Zhou et al., obtained similar results with an R0 between 2.8 and 3.3 [6]. Lipsitch et al. reported that severe acute respiratory syndrome-associated coronavirus (SARS-CoV) had an R0 value of approximately 2.2 to 3.6 [7]. The transmission capacity of COVID-19 appeared to be similar or higher as compared with SARS-CoV. As a continuous unconventional public health event that can lead to economic recession and health damage, the government’s decision making should strike a balance among the prevention and control of the epidemic and the economy and people’s livelihood. After the occurrence of the epidemic peak, it is time to gradually implement the decision to resume work and production. Hence, it is of great importance to timely judge the peak of the epidemic and determine if it is possible to return to normal work and life. Faced with such a sudden outbreak of a new infectious disease, we have no history to learn from and little evidence to refer to. The traditional epidemic peak prediction models are highly sensitive to the pre-specified epidemiological parameters including R0, and the prediction results can be highly inconsistent due to different assumptions and data sources. One study obtained two results based on two different assumptions about R0. If R0 was assumed to be higher than 1 continuously, the number of infection cases would continue to rise by the end of February, in China. However, if it was assumed to decline gradually due to public health policies, then, the epidemic peak would occur in late February [8]. Other researchers have used early epidemiological parameters to predict that the turning point of the COVID-19 epidemic, in China, would occur on 26 January [9]. Another study used data as of 12 February to make a prediction. The result suggested that the number of newly confirmed cases in China (excluding Hubei Province) and in Hubei Province (excluding Wuhan City) would both peak approximately on 1 or 2 February [10]. There are significant differences between the results of these studies, indicating the uncertainty of traditional peak prediction methods. The traditional models have proven to be most reliable only under ideal conditions.

To eliminate these limitations and build a practical index based on public reported data to timely evaluate the current epidemic situation, and then judge the epidemic peak robustly, we used the moving average of log-transformed daily new cases (LMA) to establish a specific index named “epidemic evaluation index” (EEI). The EEI was verified through previous SARS epidemic data in 2003, and then adopted to analyze the present COVID-19 epidemic based on its daily change.

## 2. Materials and Methods

We obtained the epidemic data in China available from 16 January to 20 April 2020 in the Chinese public domain [11,12], the data outside China from 27 January to 20 April 2020 on the WHO website [2], and the data for the USA from 20 February to 19 April 2020 on the U.S. CDC website [13]. On the basis of the publicly reported data, we calculated two indicators to timely judge the epidemic peak and to determine the time to resume production. Since the COVID-19 epidemic has not lasted a full year, it was difficult to judge whether the current season included the highest incidence. Therefore, the peaks we judged were all considered to be local epidemic peaks. All statistical analyses were carried out using SAS 9.4.

### 2.1. MovingAaverage of Log-Transformed Daily New Cases (LMA)

The pneumonia caused by COVID-19 has a median incubation period of 3.0 days [4]. According to the NHC (National Health Commission of the People’s Republic of China), the average time from onset to diagnosis was 4.95 days by 17 February 2020, therefore, the number of new cases per day could not accurately account for the epidemic situation. The choice of the LMA time interval can be changed based on the dynamics of the epidemic. Here, we mainly used the 7-day LMA, which could more effectively cover both the incubation period and the duration from the first symptoms to diagnosis. Other MAs (5 day, 10 day, and 14 day) were used for sensitivity analysis and auxiliary display was performed in Appendix A.

In order to make the LMA computable, first, we added 1 to the actual observation, and then performed a natural logarithmic transformation to make the proposed index, namely EEI, more tolerant of outliers. Moreover, the index after data transformation had better stability and the interval of the index limits had an appropriate width. After data transformation, we obtained the natural logarithm of actual observation as *LN_j_*:

(1) $$LN_{j}=\ln\left(N_{j}+1\right)$$

Thus, the LMA was read as the average of *LN_j_*:

(2) $$\mathrm{LMA}=\frac{LN_{1}+LN_{2}+\cdots LN_{n}}{n}.$$

*N1*, *N2*, …, *Nn* indicates the daily new cases for the past n days (e.g., 7-day LMA on 7 February = (Sum of log-transformed daily new cases on 1 February to 7 February)/7)

When analyzing the epidemic situation day-by-day, actual observations were presented in logarithmic transformation form and the LMA curve is shown in the figures.

### 2.2. Epidemic Evaluation Index (EEI)

We predicted the COVID-19 epidemic trend in different districts of China based on the moving average and its prediction limits [14]. Furthermore, we constructed a new time-varying index named “EEI” to more intuitively reflect the epidemic situation day-by-day which helped to judge the time of the epidemic peak. The definition of the EEI indicator itself is the ratio of the number of daily new cases on two consecutive days. It is defined by the following formula:

(3) $$\mathrm{EEI}=\frac{LN_{t}}{LN_{t-1}}$$

*LN_t_* and *LN_t−_*, respectively, represents the log-transformed value of daily new confirmed cases on day t and day t-1. However, the EEI itself only indicates the epidemic situation on a certain day but cannot reflect the changes in the epidemic situation over a period of time. Therefore, instead of using the EEI itself, we used the EEI(t) as the several days’ mean of EEIs on day t to depict the curve and judge the epidemic peak based on the incubation period and the duration from the initial symptoms to diagnosis (e.g., 7-day mean of EEIs for COVID-19).

The calculation of the mean and the variance of the EEIs involved a series of parameters. Taking the COVID-19 epidemic as an example, the selected time interval is 7 days. The specific meanings of the parameters involved in the following formulas are as follows:

1. LMA_t_ indicates LMA on a certain day, namely day t (e.g., 8 April, 7-day LMA for COVID-19);
2. LMA_t−1_ indicates LMA for the previous day, namely day t−1 (e.g., 7 April, 7-day LMA for COVID-19);
3. X_t_, LN_t−6_ to LN_t_, represents log-transformed values of daily new cases on day t−6 to day t (e.g., 2–8 April);
4. X_t−1_, LN_t−7_ to LN_t−1_, represents log-transformed values of daily new cases on day t−7 to day t−1 (e.g., 1–7 April).

Through the above parameters, the mean and variance of the EEIs are calculated on the basis of Taylor expansion [15] as:

(4) $$\mathrm{EEI}\left(t\right)=\mathrm{Mean}\;\mathrm{of}\;\mathrm{the}\;\mathrm{EEIs}\approx \frac{LMA_{t}}{LMA_{t-1}}-\frac{Cov\left(X_{t},X_{t-1}\right)}{{\left(LMA_{t-1}\right)}^{2}}+\frac{Var\left(X_{t-1}\right)LMA_{t}}{{\left(LMA_{t-1}\right)}^{3}}$$

(5) $$\begin{array}{c} \mathrm{Variance}\;\mathrm{of}\;\mathrm{the}\;\mathrm{EEIs}\approx \frac{{\left(LMA_{t}\right)}^{2}}{{\left(LMA_{t-1}\right)}^{2}}\left[\frac{Var\left(X_{t}\right)}{{\left(LMA_{t}\right)}^{2}}-2\frac{Cov\left(X_{t},X_{t-1}\right)}{LMA_{t}*LMA_{t-1}}+\frac{Var\left(X_{t-1}\right)}{{\left(LMA_{t-1}\right)}^{2}}\right]\\ \left(\mathrm{Var}\;=\;\mathrm{Variance},\;\mathrm{Cov}\;=\;\mathrm{Covariance}.\right) \end{array}$$

Then, the upper and lower limits of the EEI on day t, which were used as auxiliary parameters to judge the epidemic peak, was calculated using the following formula. The formula evolved from the formula for calculating prediction limits of MA [16] as:

(6) Index limit of the EEI on day t=EEI(t)±1.96×√Variance

A line chart of the EEI(t) over date indicates the epidemic situation. If the EEI(t) is higher than 1.0, it suggests that the epidemic is still developing, and the peak has not occurred. High level restriction measures should be maintained. If the EEI(t) drops below 1.0 for the first time, it can be judged that the epidemic has peaked and it is time to plan to phase out some stringent preventive and control measures. If the EEI(t) remains below 1.0 for more than 7 days (one of the time intervals of the LMA), it indicates that the epidemic has entered a stable remission period. It is possible to phase out highly stringent restrictions, and then resume production gradually. The upper and lower limits of the EEI on day t are auxiliary parameters for evaluating the epidemic situation and represent the worst and the best estimates of the epidemic situation, respectively. If the upper limit of the EEI on day t is maintained not far from 1.0 and at a relatively stable level, it indicates that the epidemic has stabilized. Moreover, if the upper limit of the EEI is less than 1, it indicates that under the worst circumstance, the EEI is also at a low level. It can be said with greater confidence that the epidemic would soon be completely eliminated. Likewise, if the lower limit of the EEI is above 1.0, this indicates that the epidemic is still in a severe period of progress. If there are very few daily new confirmed cases (<5), the EEI(t) would not be applicable. In this scenario, we should evaluate the epidemic situation based on suspected cases or other indicators.

### 2.3. The Verification of EEI(t)

SARS epidemic data for Hong Kong in 2003 was used to verify the practicability of the EEI(t). The mean incubation period of SARS was 5 to 7 days [17] and the average duration of onset of symptoms to hospital admission was 3.8 days [18], therefore, we used the 10-day LMA and the corresponding ratio to verify that the result was consistent with the actual epidemic peak.

## 3. Results

### 3.1. The Verification of the New Index

In our research, we adopted a new parameter “EEI(t)” to judge the COVID-19 epidemic peak. The EEI(t) is easier to understand than the traditional prediction models, but it has not been tested. To verify the practicability of the new index, we obtained the epidemiological data for the Hong Kong Special Administrative Region from 17 March to 21 May 2003 available on the WHO website [19] and from the appendix of a Chinese study [20].

**Figure 1:**
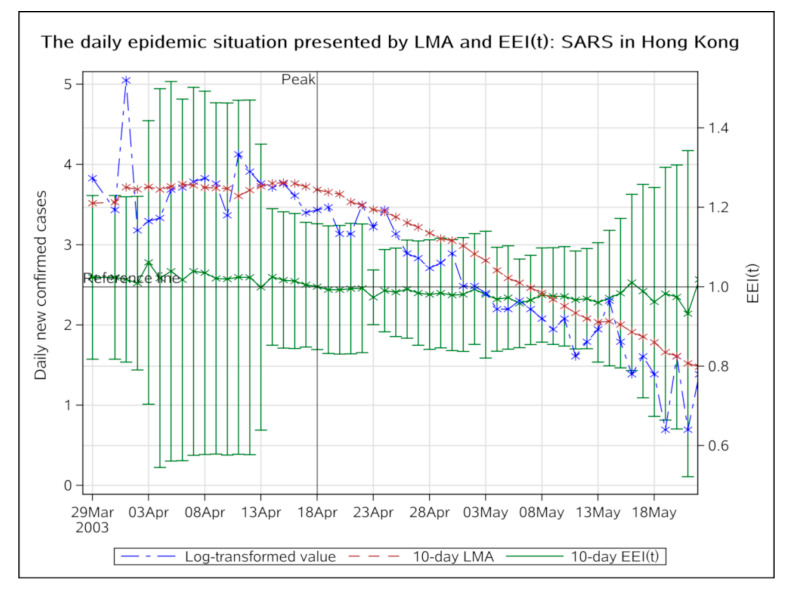
The daily epidemic situation presented by LMA and EEI(t), SARS in Hong Kong, from 29 Mar to 21 May 2003. LMA, moving average of log-transformed daily new cases and EEI(t), epidemic evaluation index (t).

The blue curve which represents the change in log-transformed value of actual observation is highly undulating, making it difficult to evaluate the trend. The EEI(t) is more stable and intuitive (Figure 1). The epidemic peak can be judged by directly comparing the index with 1.0. From 11 May 2020, the daily new confirmed cases had dropped below 5, therefore, the EEI(t) was no longer applicable. On the basis of the number itself, was determined that the epidemic was in its final phase. The fluctuations at the end of the epidemic were negligible at this time. From 18 April 2020, the EEI(t) had declined below 1.0, suggesting the epidemic had peaked. We can see that from the same day (18 April 2020) to the end, the overall SARS epidemic trend had been indeed declining, which verified the conclusion indicated by the EEI(t). The SARS epidemic in Hong Kong peaked on 18 April 2020.

In Hong Kong, SARS began to erupt and the number of cases increased sharply approximately on 29 March 2003 [21]. From 26 March 2003, the local government required close contact cases to go to designated hospitals for examination, and from 29 March 2003, all schools were closed for two weeks. About three weeks (three times the incubation period) later, i.e., on 18 April 2003, the epidemic had peaked. The peak of the epidemic coincided with the actual measures of epidemic prevention and control.

According to previous estimates of the SARS epidemic peak in Hong Kong, different studies have yielded different results. Peiris suggested that on 25 April 2003, the epidemic in Hong Kong showed signs of peaking [22], whereas Lee concluded that the epidemic peaked at the end of March [23] and Xu estimated that the epidemic would peak 14 days (on 14 April) after the start date (on 31 March) [20]. It can be seen that the conclusions of previous prediction models were quite different, and it was difficult to determine which one had a higher degree of reliability when it was applied to an epidemic peak prediction. However, now, we can see that the SARS epidemic peaked on 14 April, which was the hypothesis closest to the actual situation, and also the hypothesis closest to our conclusion.

The result indicated by the EEI(t) is verified by the overall SARS epidemic situation and is consistent with actual progress of measures for epidemic prevention and control. These two aspects show that the reliability of the EEI(t) is the same as the above-mentioned peak prediction methods and is consistent with the actual situation. The index we proposed seems realistic and acceptable.

### 3.2. Judgement of the COVID-19 Epidemic Peak

We judged the COVID-19 epidemic peak according to different regional levels. Daily new cases were mainly calculated in a laboratory-confirmed category. If there are very few daily new confirmed cases (<5), a suspected cases category was also considered. The epidemic broke out on different dates in different districts, therefore, the start dates, in the following figures, are different for each region.

#### 3.2.1. China

##### Hubei Province

**Figure 2:**
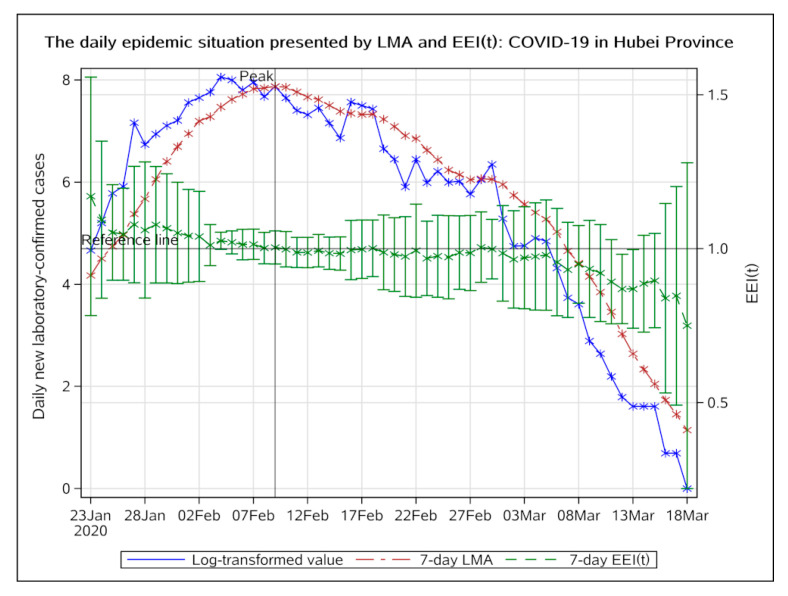
The daily epidemic situation presented by LMA and EEI(t), COVID-19 in Hubei Province, from 23 January to 18 March 2020. The blue line indicates the change of the log-transformed value over date. The brown line indicates the change of 7-day LMA over date. The green line indicates the change of the EEI(t) over date. LMA, moving average of log-transformed daily new cases and EEI(t), epidemic evaluation index (t).

Hubei Province accounts for the majority of cases in China, therefore, the epidemic situation is the most severe. On 9 February 2020, both the log-transformed value of actual observation and seven-day LMA started to decline (Figure 2).

Since 10 February 2020, the EEI(t) has been less than 1.0. We can see that the index was slightly higher than 1.0 on 18 February and 28 February, because the NHC data included not only laboratory-confirmed cases but also clinically diagnosed cases, during days before and after. We consider it to be a normal fluctuation. If public health measures prove to be effective, the epidemic should not usher in a second peak. The epidemic in Hubei Province peaked on 9 February. The epidemic entered a period of decline on 9 February and entered a stable remission period on 17 February. Before 18 March, the EEI(t) did not rebound. After 18 March, only three new laboratory-confirmed cases occurred, indicating that there was no epidemic of imported cases in Hubei Province by the current date.

##### Provinces Outside Hubei

**Figure 3:**
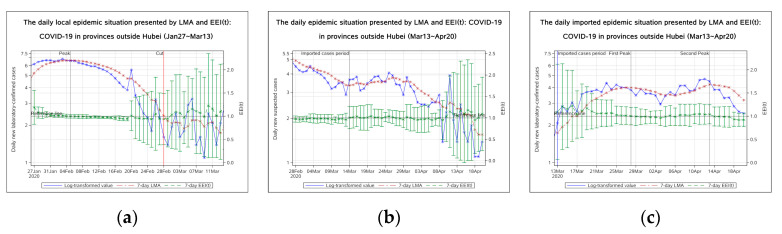
The daily epidemic situation presented by LMA and EEI(t), COVID-19 in provinces outside Hubei. (**a**) From 27 January to 13 March 2020; (**b**) From 28 February to 20 April 2020; (**c**) from 13 March to 20 April 2020. The blue line indicates the change of the log-transformed value over date. The brown line indicates the change of 7-day LMA over date. The green line indicates the change of the EEI(t) over date. LMA, moving average of log-transformed daily new cases and EEI(t), epidemic evaluation index (t).

The log-transformed value of daily new laboratory-confirmed cases peaked on 3 February 2020. The seven-day LMA has declined since 5 February in provinces outside Hubei (Figure 3a).

The peak in provinces outside Hubei occurred earlier than Hubei Province. The EEI(t) declined from 4 February 2020 and was below 1.0 on 6 February. In provinces outside Hubei, the epidemic peaked approximately on 5 February. The epidemic entered a period of decline on 5 February and entered a stable remission period on 13 February. It is worth noting that the upper limit of the EEI fell below 1.0 on 13 February. However, after a period of decline, the index suddenly rose to over 1.0 again, because of the massive growth of daily new laboratory-confirmed cases on 20 February and on 26 February. The rise on 20 February was caused by cluster epidemics in prisons. On 28 February, the number of daily new laboratory-confirmed cases dropped to less than 5, therefore, we replaced the EEI(t) of laboratory-confirmed cases with the EEI(t) of suspected cases to evaluate the epidemic situation after 28 February (red vertical bar “cut” in Figure 3a).

On 14 March 2020, the EEI(t) of daily new suspected cases rose above 1.0 (Figure 3b), which indicated that a new epidemic period caused by imported cases had begun. The imported epidemic experienced two peaks, respectively, on 28 March and 13 April, indicated by the change of the EEI(t) of daily new laboratory-confirmed cases during the imported cases period (Figure 3c). From the end of March, the imported epidemic was brought under control once. However, the open port Suifenhe City on the Sino-Russian border led to another growth of imported cases in early April. On 13 April, the imported epidemic became moderate again. It shows that the imported epidemic has been fluctuating and the prevention and control measures regarding the entry and exit management need additional strengthening. The imported epidemic has not entered a stable remission period.

#### 3.2.2. Areas outside China

**Figure 4:**
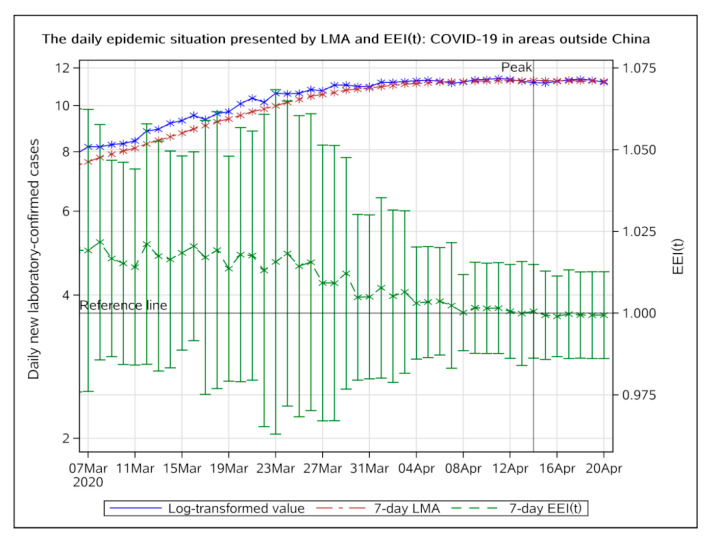
The daily epidemic situation presented by LMA and EEI(t), COVID-19 in areas outside China, from 7 March to 20 April 2020. The blue line indicates the change of the log-transformed value over date. The brown line indicates the change of 7-day LMA over date. The green line indicates the change of the EEI(t) over date. LMA, moving average of log-transformed daily new cases and EEI(t), epidemic evaluation index (t).

Figure 4 shows that the epidemic, outside China, peaked on 14 April 2020. The development of the epidemic, outside China, has been controlled to a certain extent due to prevention efforts made by countries all over the world.

The main countries with outbreaks should continue to take effective measures to further accelerate the end of the global epidemic. Here, we take the Republic of Korea and Singapore in the West Pacific Region, Italy and Germany in the European Region, Iran in the Eastern Mediterranean Region, and the USA in the region of the Americas as examples to evaluate the daily epidemic situation and judge the epidemic peak.

##### West Pacific Region

**Figure 5:**
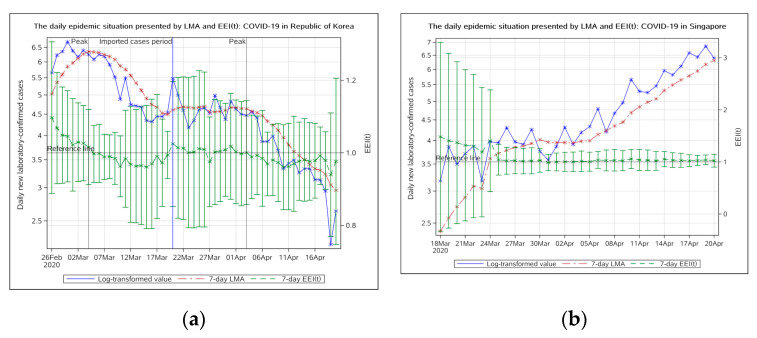
The daily epidemic situation presented by LMA and EEI(t), COVID-19 in the Republic of Korea (**a**) and Singapore (**b**). The blue line indicates the change of the log-transformed value over date. The brown line indicates the change of 7-day LMA over date. The green line indicates the change of the EEI(t) over date. LMA, moving average of log-transformed daily new cases and EEI(t), epidemic evaluation index (t).

In the Republic of Korea, the local epidemic peaked on 4 March 2020 (Figure 5a). The epidemic entered a period of decline from 4 March and entered a stable remission period from 12 March. On 20 March, the EEI(t) rose above 1.0 again, due to imported cases. The imported epidemic in the Republic of Korea peaked on April 3 and remained below 1.0. It entered a period of stable remission on 11 April 2020.

Singapore had always been regarded as one of countries whose epidemic was stable. But on 7 March 2020, the daily new cases rose to above 10 for the first time. After that, the epidemic appeared to become severe. The EEI(t) has not dropped below 1.0 by the current date (i.e., 20 April 2020), indicating that the epidemic in Singapore has not peaked and has continued to develop (Figure 5b).

##### European Region

**Figure 6:**
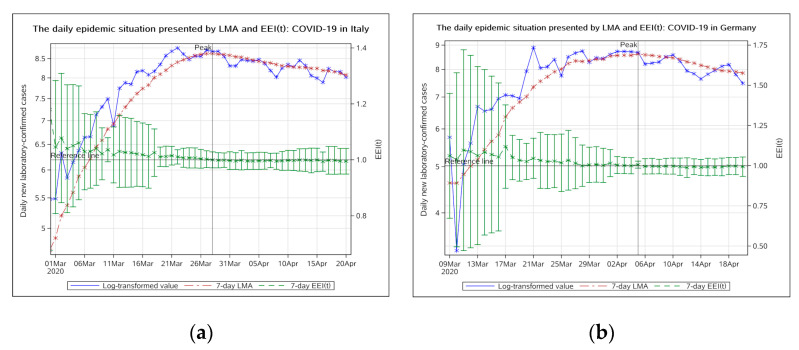
The daily epidemic situation presented by LMA and EEI(t), COVID-19 in Italy (**a**) and Germany (**b**). The blue line indicates the change of the log-transformed value over date. The brown line indicates the change of 7-day LMA over date. The green line indicates the change of the EEI(t) over date. LMA, moving average of log-transformed daily new cases and EEI(t), epidemic evaluation index (t).

In Italy and Germany, the EEI(t) fell below 1.0 on 29 March and 6 April 2020, respectively, indicating that the epidemic, in these two countries, has already peaked. In Italy, the epidemic peaked on 28 March and entered a stable period of remission on 5 April (Figure 6a). In Germany, the epidemic peaked on 5 April and entered a stable period of remission on 13 April (Figure 6b).

##### Eastern Mediterranean Region

**Figure 7:**
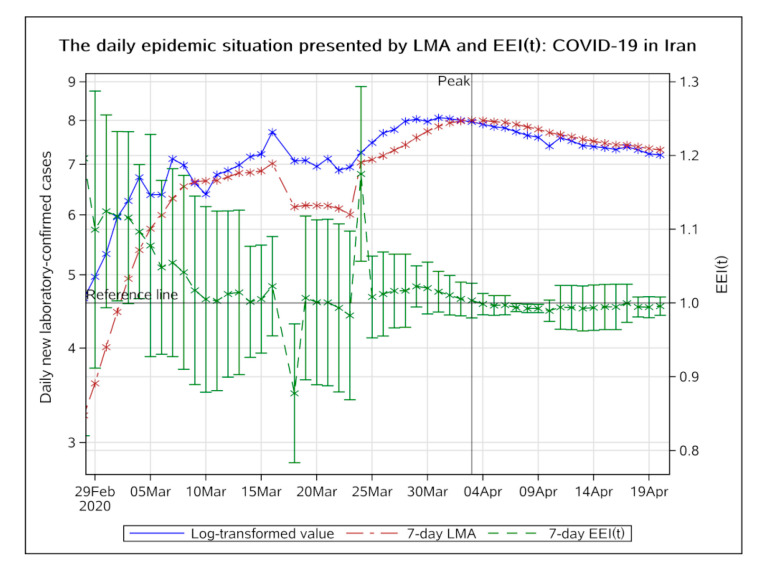
The daily epidemic situation presented by LMA and EEI(t), COVID-19 in Iran. The blue line indicates the change of the log-transformed value over date. The brown line indicates the change of 7-day LMA over date. The green line indicates the change of the EEI(t) over date. LMA, moving average of log-transformed daily new cases and EEI(t), epidemic evaluation index (t).

In Iran, the epidemic appeared to be unstable at the initial stage. The EEI(t) fluctuated for a long time and finally dropped below 1.0 on 3 April 2020, indicating that the epidemic peaked on 3 April (Figure 7). On 11 April, it entered a stable remission period.

##### Region of the Americas

**Figure 8:**
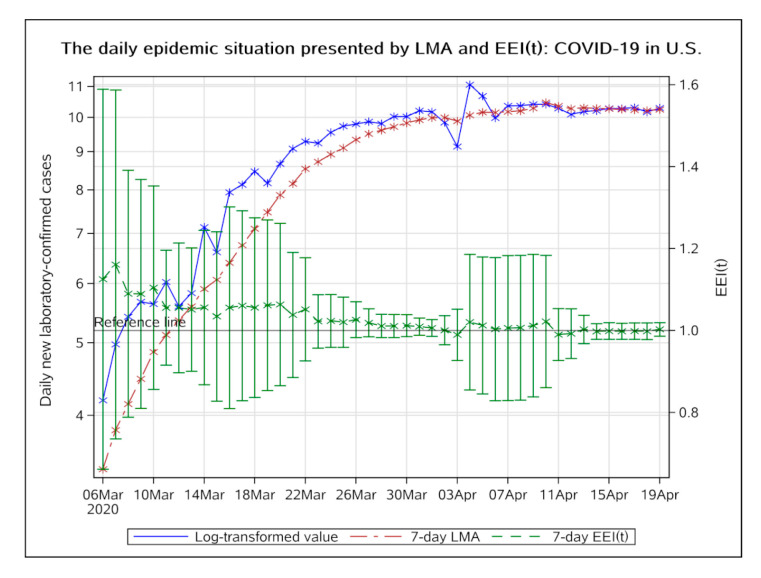
The daily epidemic situation presented by LMA and EEI(t), COVID-19 in the USA. The blue line indicates the change of the log-transformed value over date. The brown line indicates the change of 7-day LMA over date. The green line indicates the change of the EEI(t) over date. LMA, moving average of log-transformed daily new cases and EEI(t), epidemic evaluation index (t).

In the USA, the EEI(t) dropped below 1.0 on 13 April 2020 but rose above 1.0 again on 19 April (Figure 8). Subsequent changes are still needed to be observed. It can be expected that the peak of the epidemic in USA should occur soon based on continuous strengthening of preventive measures.

## 4. Discussion

A comparison with the EEI(t) curve shows that the actual observation curve always fluctuates greatly and makes it difficult to judge the epidemic peak. The decline in the log-transformed actual observation curve does not mean that the peak has occurred, as it can rise again. The EEI(t) can more accurately reflect the true situation of the epidemic. It aims to judge whether the epidemic peaks occurred or not, based on the combination of actual observations and LMA.

Traditional models, including Susceptible-Exposed-Infectious-Recovered (SEIR) and auto regressive integrated moving average (ARIMA) models, require preset application conditions. The new proposed EEI(t) avoids these limitations and is more intuitive and reliable with only officially released data. The EEI(t) aims to monitor and evaluate the epidemic in time, not to make predictions. It adopts another idea to provide timely decision support for epidemic prevention strategies. On the basis of the analysis of the epidemic situation, it can be seen that the EEI(t) has certain reliability for judging the epidemic peak.

Regarding the COVID-19 epidemic situation, it can be concluded that whether in Hubei Province or other districts, the epidemic entered a period of decline in late February due to many timely measures that were taken. In 2003, public health measures including individual isolation, quarantine, social distancing, and community containment played decisive roles in controlling the SARS epidemic [24]. During the COVID-19 epidemic, the Chinese government learned lessons from the experience of the SARS epidemic and adopted emergency measures related to population prevention and clinical treatment after the outbreak. A comparison with the prevention and control of the previous SARS epidemic shows that significant progress has been made during the COVID-19 epidemic [25]. On 20 January 2020, 43 days after the first unexplained pneumonia case occurred, traffic control was implemented in Wuhan. Three days later, on 23 January, Wuhan city was closed. Two days later, on 25 January, all provinces in China, except Tibet, triggered a level one public health emergency response. As can be seen from Figure 2 and Figure 3, the peak of daily new laboratory-confirmed cases in Hubei province was on 9 February, approximately 14 days (one quarantine observation period) after the public health emergency response, which indicates that the public health measures proved to be effective. The peak in other provinces appeared earlier, approximately on 5 February. A possible reason for this was that the epidemic outside Hubei Province was less serious and easier to control. We can see that the first-grade prevention policy (on 20 January) had not been carried out until 20 days after the outbreak (on 31 December). After the SARS epidemic in 2003, China’s public health system has received attention, but problems that emerged during the initial stage of the COVID-19 epidemic indicated that it still lacks the ability to respond quickly to unexpected infectious diseases and public health emergencies. There is still much room for improvement in the prevention and control system for major public health emergencies [26].

The EEI(t) in China has fallen below 1.0. In order to avoid excessive economic and social burden caused by the epidemic restriction measures, localities should take actions according to current epidemic situation. In Hubei Province, as the initial district of the epidemic outbreak, the EEI(t) has risen to slightly more than 1.0 twice. It suggests that after the epidemic peak, Hubei Province had to go through a long transition period before entering the final end phase. The local government planned to restore production of some enterprises and planned to normalize residents’ travel and public transportation after the peak (9 February 2020). After 17 February, the government reduced the restriction level and gradually restored urban traffic. In provinces outside Hubei, the EEI(t) of daily new laboratory-confirmed cases experienced two brief increases, one of which was caused by the prison epidemic. The upper limit of the index had been below 1.0 for several consecutive days which indicated that the speed of the epidemic to the end in areas outside Hubei was faster than in Hubei Province. However, resumption of work and return trips could lead to an increase in new cases. For example, on 26 February the index temporarily rose above 1.0. Local governments, in these districts, could gradually withdraw highly tight restrictions and public health emergency response based on the low-risk level of epidemic after 13 February. At the same time, public places and enterprises should continue to monitor body temperature. If public places such as department stores and restaurants are to be opened, disinfection measures should be emphasized. On 14 March, a new epidemic period caused by imported cases began. Due to the development of the epidemic worldwide, the direction of domestic epidemic prevention must be changed to prevent the spread of imported cases.

Outside of China, the EEI(t) fell below 1.0 on 14 April 2020, indicating that the global epidemic had been controlled to a certain extent. The epidemic outside China peaked on 14 April. Countries with a rapidly increasing number of confirmed cases can learn from China’s prevention and control experience and integrate it with their own economic situation and sociocultural background. Governments of the countries which have experienced epidemic peaks can begin to resume production, while continuing to focus on disinfection measures and maintain social distance, in order to accelerate the end of the global epidemic.

Measures taken by the Republic of Korea, Italy, Germany, and Iran to control the epidemic have shown effectiveness according to the EEI(t). On 23 February 2020, the Republic of Korea raised the level of COVID-19 epidemic warning to the highest level. Ten days later, on 4 March, the epidemic reached its peak. On 10 March, Italy closed the whole country and 18 days later, on 28 March, the epidemic reached its peak. The epidemic, in Germany and Iran, has experienced a long period before the occurrence of the peaks. In all these four countries, the epidemic has reached a stable remission period, and therefore some of the isolation measures could be withdrawn. However, on 20 March, the EEI(t) in the Republic of Korea rose above 1.0 due to imported cases, indicating that unless the epidemic in all countries is alleviated, no country can alone avoid the potential threat of the epidemic. In Singapore, the EEI(t) has not dropped below 1.0, and in the USA, the EEI(t) was still unstable and has not been continuously below 1.0. Further efforts are needed to eventually control the epidemic. Another issue worthy of attention is that the EEI(t) dropped below 1.0 twice, but then quickly returned to above 1.0 in the USA. This phenomenon suggests that a confirmation period of two to three days could be required to finalize the epidemic peak through the EEI(t). Local governments should combine the theoretical basis and the actual situation.

## 5. Conclusions

In conclusion, the EEI(t) we propose has good stability and application value and it can be used to provide evidence for the resumption of production and prevention and control measures. According to the results indicated by the EEI(t), it can be inferred that, in China, the local epidemic peaked in early February and has come to an end. However, disinfection, isolation, personnel registration, and other prevention measures must not be stopped. The focus of China’s epidemic prevention should shift to imported epidemic. The practice of China in preventing and controlling the COVID-19 epidemic has offered an important lesson to other countries. In areas outside of China, many countries are still facing challenges in controlling the epidemic and orderly resumption of work after the epidemic. The occurrence of the epidemic peak is crucial for determining economic and social recovery. The present epidemic situation in countries including the Republic of Korea, Italy, Germany, and Iran indicates that the epidemic can be contained if efficient prevention and control measures are taken as soon as possible. Although the global epidemic has shown significant relief and peaked in the middle of April, as long as the epidemic is uncontrolled anywhere in the world, we cannot fully return to normal life. As Bill Gates puts it, “no one could predict what the chance of a new virus emerging was” [27]. This is an unprecedented situation. This is a common enemy against humanity at this point. The only way we can defeat this pandemic is through solidarity [28]. All countries in the world should work together through international cooperation and take timely actions to slow and stop the spread of COVID-19 and take control of the novel coronavirus pandemic.

## Figures and Tables

**Figure A1:**
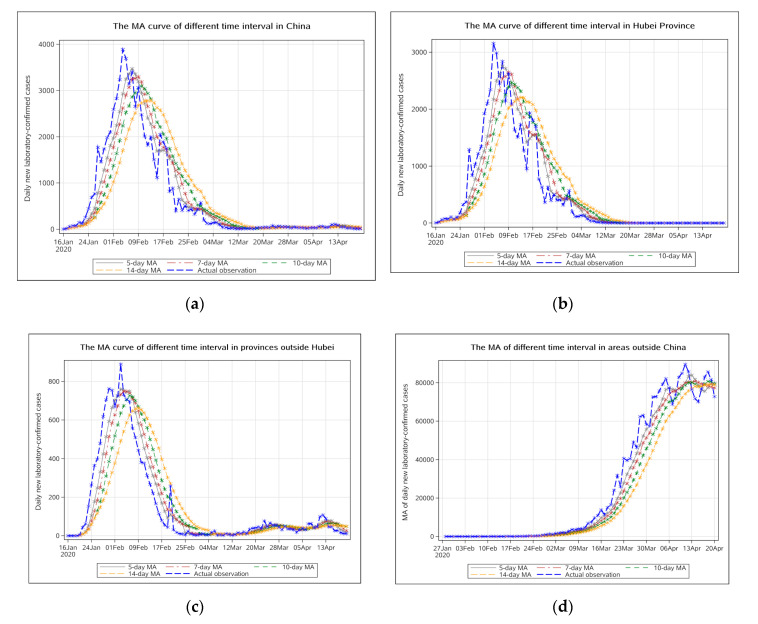
The 5-day, 7-day, 10-day, and 14-day MA of daily new laboratory-confirmed cases, from 16 January to 20 April 2010. (**a**) In China; (**b**) In Hubei Province; (**c**) In provinces outside Hubei; and (**d**) In areas outside China.

## Appendix A

Sensitivity analysis was performed using different MAs (5 days, 10 days, and 14 days). When comparing MA curves of different time intervals, MA is presented in its actual value. The 5-day, 7-day, 10-day, and 14-day MAs of daily new laboratory-confirmed cases are presented below (Figure A1). A comparison of MA curves of different durations shows their respective characteristics.

Figure A1 shows that all curves in China and in areas outside China have experienced the turning points. It can be inferred that the epidemic both peaked in China and outside China.

A comparison of different MA curves indicates that the longer the interval, the later the peak appeared. The 14-day MA curve appears to be the most stable curve and the 5-day MA curve is the most unstable curve. We can use the MA of different time intervals to evaluate trends based on the current public health strategy and the urban traffic situation at different stages. Regardless of the time interval of the MA, the peak of the epidemic in provinces outside Hubei always appears earlier than that in Hubei Province, which is consistent with our conclusion based on the 7-day LMA.
